# Polydatin Alleviates Cyclophosphamide-Induced Mouse Immunosuppression by Promoting Splenic Lymphocyte Proliferation and Thymic T Cell Development and Differentiation

**DOI:** 10.3390/ijms26062800

**Published:** 2025-03-20

**Authors:** Na Sun, Huimin Yan, Xiuping Liu, Xingdi Xu, Wei Zhao, Jing Zhang, Meng Wang, Yuxuan Liu, Lin Miao

**Affiliations:** 1 State Key Laboratory of Chinese Medicine Modernization, Tianjin University of Traditional Chinese Medicine, Tianjin 301617, China; sn1635093846@163.com (N.S.); huiminyan7978@163.com (H.Y.); liuxiuping_1@163.com (X.L.); 18838165046@163.com (W.Z.); zj18406564217@163.com (J.Z.); biubiutyuiop@163.com (M.W.); 2 Institute of Traditional Chinese Medicine, Tianjin University of Traditional Chinese Medicine, Tianjin 301617, China; xuxingdi2021@163.com; 3 Key Laboratory of Pharmacology of Traditional Chinese Medical Formulae, Ministry of Education, Tianjin University of Traditional Chinese Medicine, Tianjin 301617, China; 4 Key Laboratory of Immune Microenvironment and Disease, Immunology Department, Ministry of Education, Tianjin Medical University, Tianjin 301617, China; safexuan@126.com

**Keywords:** polydatin, immunosuppression, splenic proliferation, cell cycle rest, cyclophosphamide

## Abstract

Immunosuppression increases disease risk, and the natural compound polydatin (PD) has been reported to modulate immune-related disorders. In cyclophosphamide-induced immunosuppressed mice, PD was evaluated for its immunomodulatory effects. Immune organ indices were measured, while H&E staining and ELISA assessed spleen pathology and serum cytokine levels. The proliferation of splenic lymphocytes, both total and subpopulation, was determined using concanavalin A or lipopolysaccharide stimulation, with flow cytometry analyzing peripheral blood and splenic lymphocytes, thymic T cell subtypes, cell cycling, and bromodeoxyuridine incorporation. Western blotting was used to assess Ki67, PCNA expression, and MAPK activation. PD significantly alleviated cyclophosphamide-induced reductions in spleen and thymus indices, improved the organization of red and white pulp in the spleen, and restored TNF-α and IFN-γ levels. It reversed cyclophosphamide-induced cell cycle arrest, characterized by increased PCNA and decreased Ki67, and corrected the diminished numbers of B and T cells and the reduced CD4^+^/CD8^+^ ratio in the thymus. In vitro, PD directly promoted splenic lymphocyte proliferation and cell cycling via MAPK activation. Overall, our findings demonstrated that PD alleviated mouse immunosuppression by activating splenic lymphocyte proliferation and re-organizing thymic T cell development and differentiation.

## 1. Introduction

The immunosuppressive state is one of the clinical manifestations characterized by low immune responses with proliferation and differentiation disorders of immune cells. In cancer patients undergoing chemotherapy or radiotherapy, immunosuppression is a common side effect [1]. The majority of the chemotherapeutic agents available are known to damage blood cell production, leading to a significant reduction in both B and T lymphocytes [2]. This reduction severely compromises the patient’s immune defense, rendering them more susceptible to bacterial and viral infections [3]. Recently, aging has become a global health issue where progressive weakening in the immune system such as T cell reduction and CD4^+^/CD8^+^ T cell ratio imbalances are considered to be main inducers and consequences [4]. Thus, searching for immunomodulatory medicines appears to be of great importance for not only disease therapy but also enhancement of aging life quantification.

The spleen is the largest secondary lymphoid organ in the body and plays a crucial role in the immune system. It serves as a major site for the aggregation of B and T cells and is responsible for filtering pathogens and aging red blood cells from the blood. The white pulp of the spleen is the primary site for the proliferation and differentiation of lymphocytes, including B and T cells. The proliferation of splenic lymphocytes directly reflects the state of cellular immunity in animals [5]. Cytokines are essential signaling molecules in the immune system, playing a central role in the activation, proliferation, differentiation, and functional regulation of immune cells.

In the context of immunosuppression, the spleen often exhibits significant pathological changes such as atrophy of the white pulp and a reduction in lymphocytes. Cytokine levels are typically diminished. Analyzing the histopathological changes in the spleen provides a direct assessment of the degree of immunosuppression and the restorative effects of therapeutic agents on the immune system. Similarly, evaluating cytokine levels helps to assess the functional state of the immune system and the efficacy of drugs in restoring the immune function.

Cell proliferation is closely linked to cell cycling, and two well-known proliferation markers, PCNA and Ki67, respond to the induction of proliferation with increased expressions [6,7]. Chemotherapeutic agents such as cyclophosphamide (CY), cytarabine, or doxorubicin disturb cell cycles and induce cell arrest in distinct phases of cycling [8]. Dysfunction of lymphocyte proliferation is observed in various immunosuppressive conditions such as HIV, where continuous antigenic stimulation leads to lymphocyte exhaustion, impairing the ability to proliferate [9]. Excessive copper exposure in Swiss albino mice promoted cell cycle arrest and cell death in immune organs, ultimately leading to immunosuppression [10].

**Figure 1:**
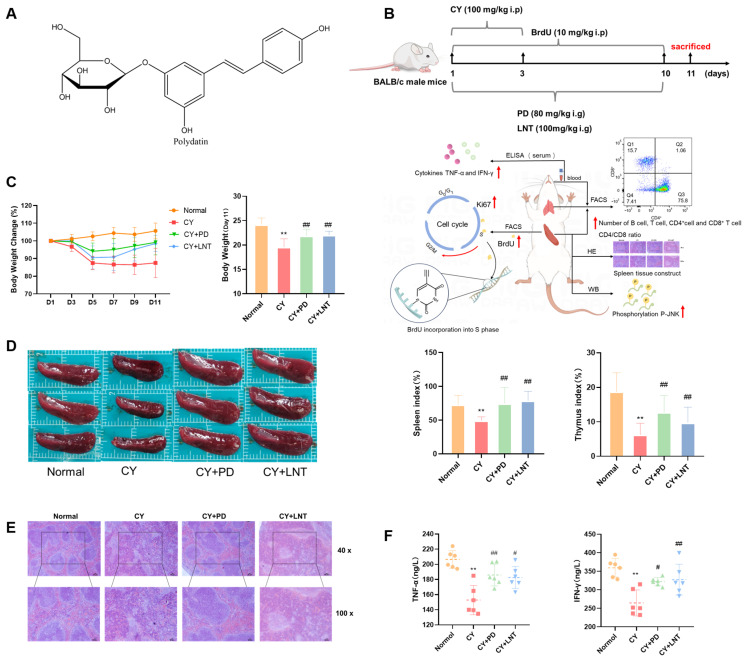
PD alleviated CY-induced immunosuppression in mice. The structural formula of PD (**A**) and the experimental treatment schedule (**B**) in vivo. Changes in body weight (*n* = 12) (**C**). Spleen and thymus indices (*n* = 12) (**D**). Representative images of spleen H&E staining (**E**). TNF-α and IFN-γ levels (*n* = 6) in serum were measured in each group (**F**). Data are expressed as mean ± SD. ** *p* < 0.01 vs. Normal group; ^#^ *p* < 0.05 and ^##^ *p* < 0.01 vs. CY group.

The natural compound polydatin (PD) is a resveratrol glucoside named as resveratrol-3-O-β-mono-D-glucoside (Figure 1A), and is abundant in *Polygonum cuspidatum Sieb. et Zucc*. As previously reported, PD exhibits high bioavailability and low toxicity [11,12]. Due to its high bioavailability, PD is rapidly absorbed through the intestines, enters the bloodstream, and is distributed to various tissues, including immune organs such as the spleen and thymus. Its stable metabolism allows it to maintain therapeutic effects over an extended period, which is particularly important for the long-term treatment of immune-related diseases such as chronic inflammation or immunosuppression. The molecular structure of PD contains a glucose moiety, enabling it to directly enter cells via active glucose carriers [13,14] Immune cells typically express high levels of glucose transporters, allowing PD to preferentially enter these cells and directly modulate intracellular signaling pathways, thereby effectively regulating the proliferation, differentiation, and function of immune cells. The cyclohexene ether skeleton of PD confers anti-inflammatory and antioxidant activities, mitigating inflammatory responses and oxidative stress-induced damage to immune cells. Moreover, a PD injection has been approved for shock treatment in phase II of clinical trials in the United States, indicating PD to be a potential candidate for drug development. As a natural plant extract, PD is widely available [15] and has minimal adverse effects [12]. It is abundant in Gentiana species, and its extraction process is well-established, allowing for scalable production. Pharmacological data show that PD, as a natural immunomodulator, exerts multi-target immunomodulatory effects, including inhibition of inflammatory mediator release [12,15,16], regulation of immune cell activity, antioxidant effects [12,16,17], and modulation of cell proliferation [18,19]. Lentinan (LNT), used as a positive control in this study, is a polysaccharide extracted from Lentinus edodes and exhibits significant immune-enhancing effects [20,21,22,23]. However, whether and how PD enhances immunity remain unclear.

Here, in our study, we aimed to investigate the immune regulatory effect of PD in CY-induced immunosuppressed mice, and uncovered whether lymphocyte proliferation and differentiation were involved.

## 2. Results

### 2.1. PD Inhibited CY-Induced Immunosuppression in Mice

To determine the immunomodulatory effect of PD (Figure 1A) in vivo, a CY-induced immunosuppression model was established in mice, and PD was administrated daily for 10 days (Figure 1B). As we expected, not only body weights but also thymus and spleen indices were dramatically decreased in the CY group when compared with mice in the Normal group (Figure 1C,D), which were significantly blocked in the PD group. A pathological analysis showed that white pulp atrophy, hemorrhage, and necrosis in the spleen were easily observed and the white red pulp was intermixed in the CY group when compared with those in the Normal group. However, after PD administration, the pathological alterations in the spleen were much alleviated (Figure 1E). Consistently, serum levels of TNF-α and IFN-γ were much reduced in CY mice, and PD significantly inhibited a CY-induced cytokine decrease (Figure 1F). LNT was used as a positive control and, consistent with previous reports, parameters including body weight, thymus, and spleen indices as well as splenic pathological staining and cytokine levels were also much recovered [22,23,24].

### 2.2. PD Promoted Splenic Lymphocyte Proliferation In Vivo and In Vitro

**Figure 2:**
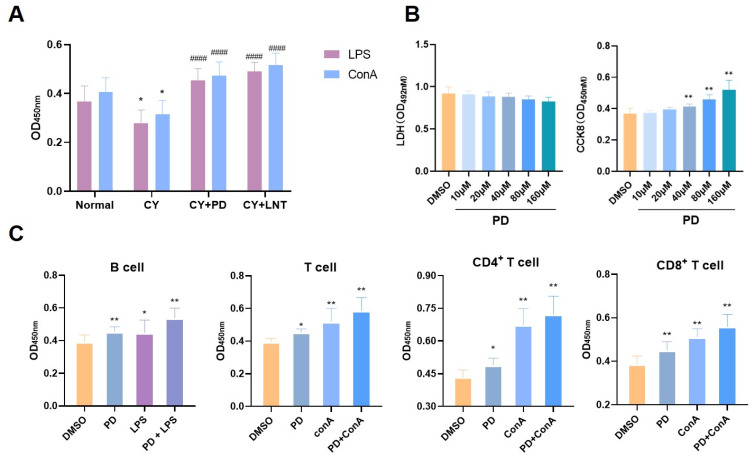
PD stimulated splenic lymphocyte proliferation in vivo and in vitro. ConA-induced T lymphocyte and LPS-induced B lymphocyte proliferation (*n* = 6) (**A**). The LDH level was analyzed using an LDH assay kit and cell proliferation was measured using a CCK-8 assay (*n* = 3) (**B**). Comparison of purity of splenocyte subsets before and after magnetic bead sorting (**C**). After stimulation with PD, the proliferation of each subset was highly increased, which was further enhanced when combined with ConA or LPS. Data are expressed as mean ± SD. * *p* < 0.05 and ** *p* < 0.01 vs. DMSO group; ^####^ *p* < 0.0001 vs. CY group.

We first isolated splenic lymphocytes from different groups in vivo. ConA and LPS, the specific stimulators for T or B lymphocyte proliferation, were directly added. As shown in Figure 2A, PD exhibited a significant stimulatory effect on lymphocyte proliferation in response to either ConA or LPS. In vitro, the LDH results showed that PD had no significant toxic effects on splenic lymphocytes and, similar to the findings in vivo, PD also promoted splenic lymphocyte proliferation (Figure 2B). To further evaluate the effect of PD on the proliferation of splenocyte subsets, a magnetic bead sorting assay was performed and highly pure cell subsets including B cells, CD3^+^ T cells, CD4^+^ T cells, and CD8^+^ T cells were obtained (Supplementary Materials). After stimulation with PD, the proliferation of each subset was highly increased (Figure 2C), which was further enhanced when combined with ConA or LPS.

### 2.3. PD Inhibited the Abnormal Percentages of Lymphocyte Subsets in the Peripheral, Spleen, and Thymus Isolated from CY-Induced Immunosuppressed Mice

**Figure 3:**
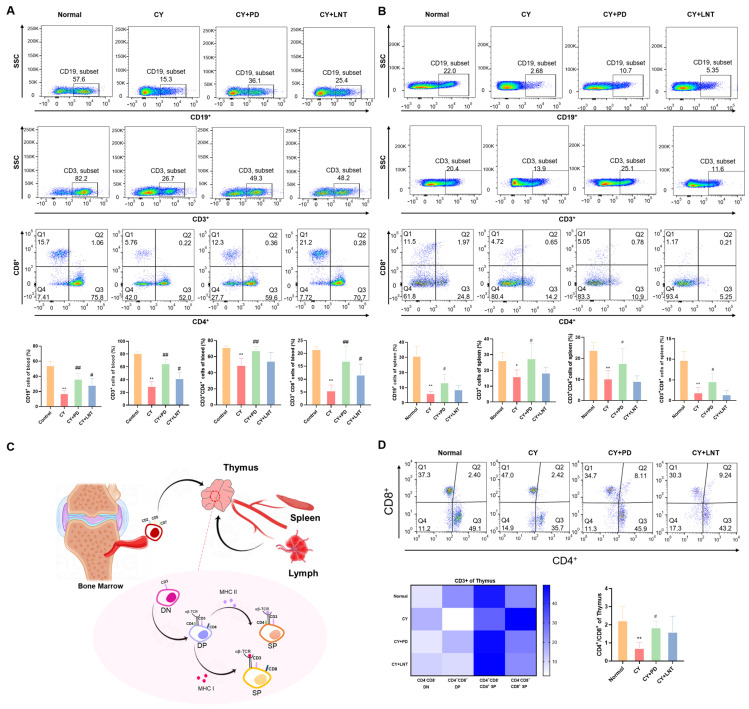
PD inhibited abnormal lymphocyte subsets in the peripheral, thymus, and spleen of CY-induced immunosuppressed mice. Cells from blood (**A**) and spleen (**B**) in Normal, CY, CY+PD, and CY+LNT groups were collected and the subsets, including B lymphocytes, T lymphocytes, CD4^+^ T lymphocytes, and CD8^+^ T lymphocytes, were measured and are shown. (**C**) Schematic representation of T cell development and migration. T cell progenitors originate in the bone marrow and migrate to the thymus, where they undergo differentiation through double-negative (DN), double-positive (DP), and single-positive (SP) stages, ultimately maturing into CD4^+^ or CD8^+^ T cells. Mature T cells then migrate to the spleen and lymph nodes, where they perform immune functions in the peripheral immune organs. (**D**) T lymphocyte subsets (CD4^−^CD8^−^, CD4^+^CD8^+^, CD4^+^CD8^−^, and CD4^−^CD8^+^) and CD4^+^/CD8^+^ in the thymus of each group and heatmap of the distribution of CD3^+^ T cell subsets in the thymus. The horizontal axis represents CD3^+^ T cell subsets and the vertical axis lists different experimental groups. The color intensity indicates the proportion of CD3^+^ T cell subsets, with darker shades representing higher proportions. Data are expressed as mean ± SD. * *p* < 0.05 and ** *p* < 0.01 vs. Normal group; ^#^ *p* < 0.05 and ^##^ *p* < 0.01 vs. CY group (*n* = 6).

T cells were classified into helper T cells (CD4^+^ T cells) and cytotoxic T cells (CD8^+^ T cells) according to their distinct antigens and function. The frequencies of lymphocyte subsets were evaluated using flow cytometry. Compared with the Normal group, the percentages of B cells, CD3^+^ T cells, CD4^+^ T cells, and CD8^+^ T cells in the peripheral blood and spleen isolated from the CY group were significantly decreased; all then recovered after treatment with PD (Figure 3A,B). T lymphocytes principally developed in the thymus (Figure 3C) [25]. Originating from shared precursors, CD3^+^ cells are initially expressed with CD4^−^ and CD8^−^ double-negative (DN) T cells, which subsequently develop into CD4^+^ and CD8^+^ double-positive (DP) T cells and selectively differentiate into SP cells (CD4^+^ or CD8^+^ T cells) [26,27,28]. As shown in Figure 3D, CY inhibited the percentages of DN but induced the percentages of DP and CD8^+^ SP T cells, leading to a significant decrease in the CD4^+^/CD8^+^ ratio. However, PD administration alleviated the interference of CY on T lymphocyte development and differentiation (Figure 3D) in the thymus.

### 2.4. PD Promoted the Proliferation of Splenic Lymphocytes in CY-Induced Immunosuppressed Mice

**Figure 4:**
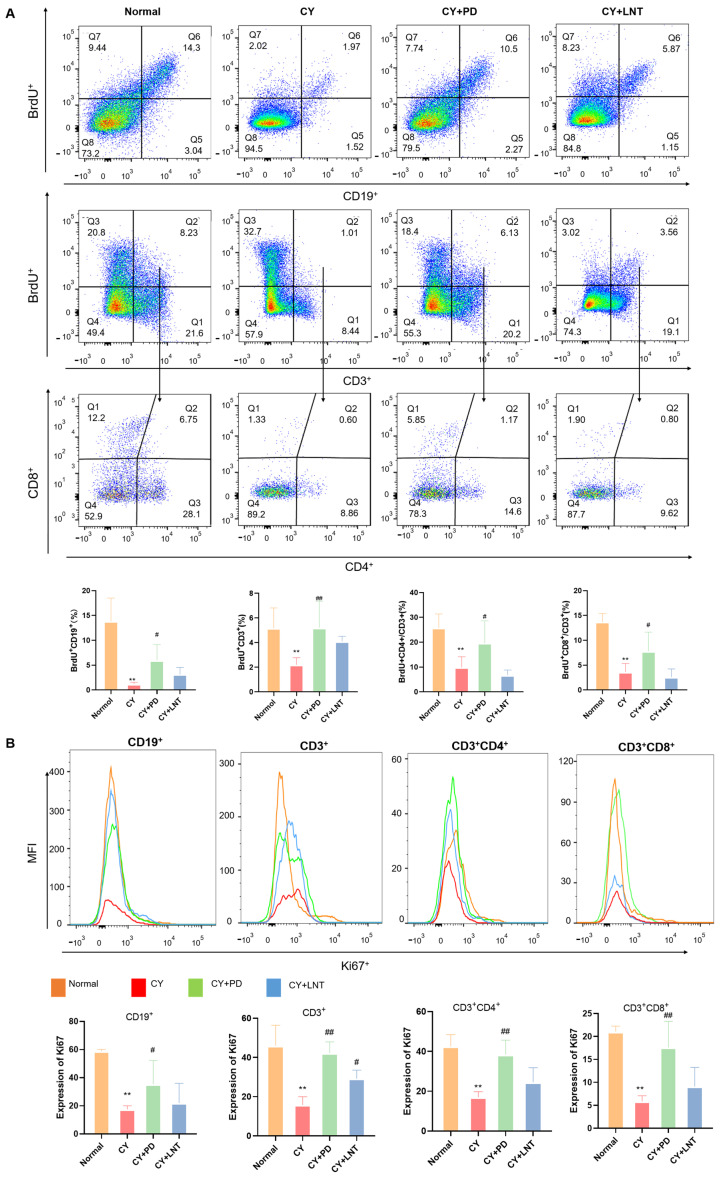
PD promoted the percentages of BrdU and expressions of Ki67 in the spleen of CY-induced immunosuppressed mice. The intensities of BrdU and Ki67 in splenic subsets were measured and are shown (**A**,**B**) for the Normal, CY, CY+PD, and CY+LNT groups. Data are expressed as mean ± SD. ** *p* < 0.01 vs. Normal group; ^#^ *p* < 0.05 and ^##^ *p* < 0.05 vs. CY group (*n* = 6).

To determine how PD induced the proliferation of splenic lymphocytes, we injected BrdU to label proliferating splenic lymphocytes in vivo. Flow cytometry was then used to detect BrdU-positive cells in the spleen. As we expected, the intensity of BrdU in splenic subsets including CD19^+^, CD3^+^, double-CD3^+^CD8^+^, and CD3^+^CD4^+^ was significantly reduced in the CY group but partly recovered after PD administration (Figure 4A). As a proliferation indicator, Ki67 expression was further investigated in splenic cells. Consistent with the BrdU data, the expressions of Ki67 in subset splenic cells were all significantly decreased in the CY group; the reduction was partially suppressed after PD treatment (Figure 4B). However, the BrdU intensity in the lymphocyte subsets of the CY+LNT group was not significantly different from the CY group. The administration of LNT exclusively facilitated the restoration of Ki67 expression in CD3^+^ T cells (Figure 4B), while the effect of LNT on lymphocyte proliferation was not statistically significant (Figure 3B).

### 2.5. PD Promoted the Restoration of Lymphocyte Numbers in the G2/M Phase

**Figure 5:**
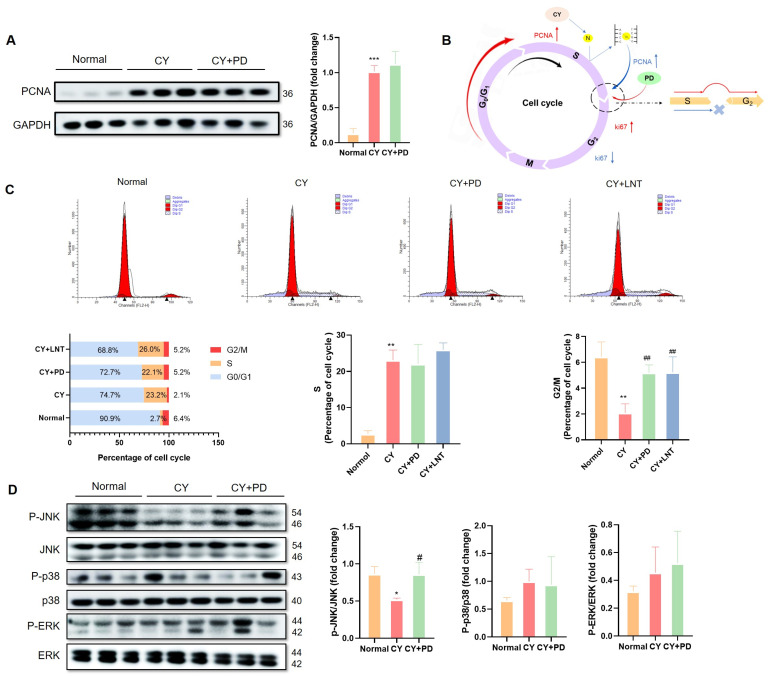
PD regulated the cell cycle and the proliferation-related MAPK/JNK pathway. Representative images of PCNA proteins were tested using a Western blot (WB) (**A**). Schematic diagram of the effects of CY and PD on cell cycle (**B**). The cell cycle in splenic lymphocytes was measured and is shown for Normal, CY, CY+PD, and CY+LNT groups (**C**). Representative images of the Western blot (WB) of phosphorylated MAPK proteins (**D**). All experiments were performed in triplicate, and molecular weight (KDa) is labeled on the right. Data are expressed as mean ± SD. * *p* < 0.05, ** *p* < 0.01, *** *p* < 0.001 vs. Normal group; ^#^ *p* < 0.05, ^##^ *p* < 0.01 vs. CY group.

As another proliferation marker, PCNA expression was detected in the spleen. In contrast to the decrease in Ki67 expression, PCNA expression in the CY and CY+PD groups was significantly increased when compared with that of the Normal group (Figure 5A). The potential mechanisms of PD on CY-induced cell-cycle arrest is summarized in Figure 5B. Compared with those in the Normal group, the splenic cells in the CY group exhibited a noteworthy increase (from 2.7% to 23.2%) in the S phase and a substantial decrease in the G2/M phase (from 6.4% to 2.1%). However, a slight inhibition in the S phase (22.1%) and a significant restoration in the G2/M phase (5.2%) were observed in the CY+PD group, both of which were comparable with those in the CY+LNT group (Figure 5C).

The MAPK cascade signaling pathways, including JNK, p38, and ERK, are involved in the regulation of cell proliferation [29]. As shown in Figure 5D, the phosphorylation level of JNK, but not of ERK and p38, was significantly downregulated in the CY group, which was then restored in the CY+PD group, suggesting that CY may inhibit splenic lymphocyte proliferation by suppressing the level of JNK phosphorylation.

## 3. Discussion

PD is a natural resveratrol derivative with high bioavailability [30] and multiple pharmacological activities such as neuroprotection [31,32], antioxidation, and anti-inflammation [33]. Previous studies have confirmed that PD can regulate the immune function [34]. In this study, we explored the effects of PD in a CY-induced immunosuppression model. CY is a widely used immunosuppressive agent that suppresses immune system function, leading to body weight loss and atrophy of the spleen and thymus organs, which are critical for maintaining immune homeostasis [35,36,37].

Our data showed that PD significantly mitigated CY-induced weight loss and reductions in spleen and thymus indices while improving the pathological state of the spleen (Figure 1B–E). Moreover, PD markedly increased the serum levels of TNF-α and IFN-γ (Figure 1F). These findings suggest that PD effectively reversed immunosuppression. It is clear that the differences in the immunomodulatory effects between the positive control group using lentinan and other studies [20,21,24,38] could be attributed to the lower dosage and shorter treatment duration of the lentinan used in the positive control group (Figure 3B).

This study demonstrated that PD significantly promoted the proliferation of splenic lymphocytes. The spleen, one of the primary lymphoid organs, plays a crucial role in the proliferation and differentiation of both B and T cells [39]. According to the literature, CY suppresses immune responses by inhibiting T and B cell proliferation [40,41]. Similarly, our research confirmed that CY significantly suppressed the basic and ConA/LPS-induced proliferation of T and B cells in the spleen (Figure 2A and Figure 3B) and the peripheral blood (Figure 3A). Simultaneously, using BrdU incorporation, we found that CY significantly downregulated the number of newly proliferating splenic lymphocytes (Figure 4A). However, the administration of PD was able to significantly reverse these effects. PD stimulated splenic lymphocyte proliferation in vivo (Figure 2A and Figure 4B) and in vitro (Figure 2B,D), and PD reversed the reduced counts of various lymphocyte subsets from the peripheral blood (Figure 3A) and spleen (Figure 3B). This result was similar to the mechanism by which polysaccharides (e.g., ginseng polysaccharides) restored CY-induced immune injury by promoting lymphocyte proliferation [42]. In addition, PD enhanced the cell proliferative capacity (Figure 4B) and increased the number of new lymphocytes (Figure 4A). However, a separate control group for CY was not established in this study, limiting the ability to directly assess the reversal of CY immunosuppression by PD.

A positive association between cell proliferation and PCNA and Ki67 expression is well known; both are considered to be proliferation markers [7,43]. It has been reported that PCNA is primarily present in the S phase of cell cycles, while Ki67 is expressed throughout the whole cell cycle [44,45,46,47,48,49]. However, our data found that the suppressive proliferation was consistent with Ki67 downregulation (Figure 4B) but also with PCNA upregulation (Figure 5A). It has previously been reported that PCNA is predominantly expressed in the S phase, which coordinates with DNA replication and repair [44,45,47,49], whereas Ki67 is expressed throughout the whole cell cycle, except the G_0_ phase. Therefore, the regulation of CY on the splenocyte cell cycle was performed and the data confirmed that CY increased the splenocyte numbers in the S phages but decreased them in the G2 phase (Figure 5C). This phenomenon suggests that CY-induced DNA damage triggered a DNA repair response in the cells, leading to the upregulation of the PCNA expression. PCNA plays a critical role in DNA replication and repair, especially during the S phase, and its increased expression indicated that the cells were more engaged in repair mechanisms in response to DNA damage. On the other hand, the reduction in Ki67 expression reflected an inhibition of cell proliferation, signifying a decreased proliferative activity. This could be attributed to the DNA damage induced by CY, which likely impeded the progression of cells from the S phase to the G2/M phase. Such a blockage aligned with cyclophosphamide’s known cytotoxic effects, ultimately preventing cells from entering the mitotic phase [36]. After PD administration, we observed an increase in the expression of both PCNA (Figure 5A) and Ki67 (Figure 4B), along with a significant rise in the proportion of cells in the G2/M phase (Figure 5C). This suggests that the treatment not only mitigated CY-induced DNA damage but also promoted the recovery of cell proliferation. The continued elevation of PCNA expression could be linked to ongoing DNA repair, while the restored expression of Ki67 indicated an enhanced cell proliferative capacity. The increased proportion of cells in the G2/M phase further supports this, showing that the treatment facilitated the successful progression of cells from the S phase to the G2/M phase, thereby promoting smooth cell cycle progression.

It is known that the p38/JNK MAPK pathway is one of the main signaling pathways involved in the promotion of cell proliferation [50], including splenocyte proliferation [51]. In this study, we first showed that CY inactivated MAPKs (Figure 5D). Further studies found that PD improved the reduction in JNK phosphorylation (Figure 5D). PD could have activated the cell cycle via activating the MAPK/JNK signaling pathway but we need to pharmacologically inhibit the MAPK/JNK pathway using a specific JNK inhibitor (SP600125, HY-12041, MedChemExpress, Monmouth Junction, NJ, USA) in subsequent stages and compare the proliferation and cell cycle progression of lymphocytes with and without the inhibitor to verify this.

In addition to splenic lymphocyte proliferation, the development and differentiation of thymic T cells are equally critical for maintaining a normal immune function. The thymus serves as the primary site for T cell development and maturation, and the differentiation of T cells in the thymus directly impacts the effectiveness of the peripheral immune system [37]. Among the immune responses, the thymus is considered to be the primary site of T cell development, differentiation, and maturation [39]. After entering the thymus, the lymphoid precursor cells undergo the following three distinct cellular states: DN cells, DP cells, or SP cells (CD4^+^ T cells or CD8^+^ T cells) [26,27,28,52]. Here, we first showed that CY inhibited the development of DN cells into DP cells and induced the downregulation of the CD4+/CD8+ ratio in the thymus (Figure 3D). However, treatment with PD effectively corrected this alteration by promoting T cell development and differentiation. This suggests that PD not only restored lymphocyte proliferation in the spleen but also reshaped T cell maturation in the thymus, thereby comprehensively enhancing the immune response. There is a close connection between splenic lymphocyte proliferation and thymic T cell development. The differentiation and maturation of T cells in the thymus supply functional T cells to the spleen, while splenic lymphocyte proliferation is a response to antigenic stimulation in the peripheral immune system. The bidirectional regulation of PD on these two critical immune organs, the thymus and spleen, indicates its comprehensive immune-enhancing effects.

Although our study has highlighted the immunomodulatory effects of polydatin (PD), it is important to contextualize these findings within the broader landscape of natural immunomodulators. For example, resveratrol, a well-known polyphenol, has been extensively studied for its anti-inflammatory and antioxidant properties. However, the low bioavailability of resveratrol limits its clinical efficacy [53,54]. In contrast, PD, as a resveratrol glucoside, exhibits higher bioavailability and more potent immunomodulatory effects, particularly in promoting lymphocyte proliferation and T cell differentiation. Similarly, lentinan, a polysaccharide derived from Lentinus edodes, has shown significant immune-enhancing effects but its mechanism primarily involves the activation of macrophages and natural killer cells [55] rather than directly modulating lymphocyte proliferation. These comparisons highlight the unique advantages of PD in terms of both its efficacy and mechanism.

The immunomodulatory effects of PD extend beyond reversing cyclophosphamide (CY)-induced immunosuppression. Our findings suggest that PD could be a promising candidate for treating other immune-related disorders such as chronic inflammation and autoimmune diseases. PD’s ability to modulate both innate and adaptive immune responses, as evidenced by its effects on cytokine levels and lymphocyte subsets, positions it as a versatile therapeutic agent. Furthermore, the low toxicity and high bioavailability of PD make it suitable for long-term use, which is particularly important for managing chronic conditions.

## 4. Materials and Methods

### 4.1. Drug Preparation

Injectable CY was obtained from Shanxi Pude Medicine Co., Ltd. (Shanxi, China). PD and lentinan (LNT) were provided by Chengdu Efa Biotechnology Co., Ltd. (Chengdu, China). 5-Bromo-2′-deoxyuridine (BrdU), lipopolysaccharide (LPS), and concanavalin protein (ConA) were provided by Bio Legend, Inc. (San Diego, CA, USA).

### 4.2. Animals

Male BALB/c mice, aged 6–8 weeks, were procured from Charles River Laboratories (Beijing, China; license number: SCXK 2016-0006) and were kept in a 12 h light and 12 h dark cycle with access to food and water. The Animal Care and Use Committee of Tianjin approved all experimental procedures (Animal Ethics Committee approval number: TCM-LAEC2023024; approval date: 14 March 2023).

### 4.3. CY-Induced Immunosuppression Model

For the in vivo assay, the mice were randomly divided into the following four groups (*n* = 12): the Normal group (Normal), the CY group (CY), the PD group (CY+PD), and the lentinan group (CY+LNT) as the positive control. For the first 3 days, the mice were intraperitoneally administered CY daily (100 mg/kg; CY, CY+PD, and CY+LNT groups) or sterile saline (Normal group). For the CY+PD or CY+LNT groups, mice were daily and continuously given PD (80 mg/kg) or LNT (100 mg/kg) by gavage for 10 days, while mice in the Normal group and CY group were intragastrically administered 0.9% (*w*/*v*) saline. To analyze the newly generated cells, BrdU incubation was conducted as previously reported [56]. Briefly, mice in all groups were intraperitoneally administered a BrdU (Sigma-Aldrich St. Louis, MO, USA; No. 59-14-3) bolus (10 mg/kg) for 10 days. Body weights were recorded every other day. After 10 days, the mice were sacrificed by administering an overdose of tribromoethanol (20 mg/kg) followed by cervical dislocation. The thymus and spleen were isolated and immediately weighed. The indices of the thymus and spleen were calculated according to the following formula: Spleen index or thymus index (%) (spleen or thymus weight/body weight) × 100%. To accomplish the following test indicators, each group was further divided into two subgroups named A and B (*n* = 6). Subgroup A was used to analyze the lymphocyte subsets of the peripheral blood (B cells, CD3^+^ T cells, CD4^+^ T cells, and CD8^+^ T cells), spleen (B cells, CD3^+^ T cells, CD4^+^ T cells, CD8^+^ T cells, BrdU intensity, and Ki67 expression), and thymus (CD3^+^ T cells, CD4^+^ T cells, and CD8^+^ T cells) using flow cytometry and HE staining for the spleens. Subgroup B was used to detect the cytokine levels in the serum (TNF-α and IFN-γ), LPS, or ConA-induced splenocyte proliferation using propidium iodide (PI) and Western blotting.

### 4.4. Hematoxylin and Eosin Staining

Spleens were fixed in 4% paraformaldehyde, embedded in paraffin, and sectioned at 4 µm intervals. These sections were then deparaffinized, stained with HE, and examined using light microscopy as previously reported [43].

### 4.5. Assessment of Serum TNF-α and IFN-γ by ELISA

Blood was collected by extracting the eyeballs of the mice and then serum was obtained by centrifugation. The levels of cytokine TNF-α (#M-TNF-96; Boaotuoda Biotechnology Co., Ltd., Beijing, China) and IFN-γ (#TOPEL03497; Boaotuoda Biotechnology Co., Ltd., Beijing, China) in the serum were measured according to the ELISA kit instructions (Boaotuoda Biotechnology Co., Ltd., Beijing, China).

### 4.6. Splenic Lymphocyte Subset Selection and Culture

Mouse spleens from male BALB/c mice were washed with cold PBS and filtered, and a homogeneous splenocyte suspension was then obtained. The erythrocytes were lysed and centrifuged at 1000 rpm for 8 min. Magnetic bead sorting kits were then used to obtain the total B cells (#721905; Precision BioMedicals Co., Ltd., Tianjin, China), CD3^+^ T cells (#720305; Precision BioMedicals Co., Ltd., Tianjin, China), CD4^+^ T cells (#722405; Precision BioMedicals Co., Ltd., Tianjin, China), or CD8^+^ T cells (#722805; Precision BioMedicals Co., Ltd., Tianjin, China) from splenic lymphocytes. Then, the cells (5 × 10^6^/mL) were seeded into 96-well plates in an RPMI-1640 medium with 20% FBS. After overnight incubation, PD (40 μM), LPS (10 μg/mL), or ConA (5 μg/mL) were added and incubated for 48 h at 37 °C in a humidified atmosphere containing 5% CO_2_.

### 4.7. Splenic Lymphocyte Proliferation and Cytotoxicity Assay

Splenic lymphocytes were obtained from male BALB/c mice and seeded in 96-well plates (5 × 10^6^/mL) in an RPMI-1640 medium supplemented with 20% FBS. After overnight incubation, various concentrations of PD (10, 20, 40, 80, and 160 μM) were added and incubated for 48 h at 37 °C in the presence of 5% (*v*/*v*) CO_2_. Then, 100 μL of LDH reagent (#SC609; DOJINDO, Kumamoto, Japan) or 10 μL of CCK-8 reagent (#35301205; YESEN, Shanghai, China) were directly added into the well (as required by the kits) and incubated for 15 min or 3 h. Absorbance at 492 nm or 450 nm was detected using a microplate reader (Infinite F50; Haitian Youcheng Technology Co., Ltd., Beijing, China).

### 4.8. Flow Cytometry

Lymphocytes from the peripheral blood, spleen, and thymus were collected from mice (Subgroup A). Subgroups of lymphocytes—including CD19^+^ B cells (#115530; BioLegend, San Diego, CA, USA), CD3^+^ T cells (#100234; BioLegend, San Diego, CA, USA), CD4^+^ T cells (#100203; BioLegend, San Diego, CA, USA), and CD8^+^ T cells (#100203; BioLegend, San Diego, CA, USA) in the peripheral blood; BrdU (#534848; Sigma, San Diego, CA, USA) or Ki67 (#652404; BioLegend, San Diego, CA, USA) in CD19^+^ B cells (#115530; BioLegend, San Diego, CA, USA), CD3^+^ T cells (#100234; BioLegend, San Diego, CA, USA), CD4^+^ T cells (#100432; BioLegend, San Diego, CA, USA), and CD8^+^ T cells (#100712; BioLegend, San Diego, CA, USA) in the spleen; and CD3^+^ T cells (#100203; BioLegend, San Diego, CA, USA), CD4^+^ T cells (#100432; BioLegend, San Diego, CA, USA), and CD8^+^ T cells (#100712; BioLegend, San Diego, CA, USA) in the thymus—were detected by incubation with appropriately diluted antibodies for 30 min at 4 °C in the dark. Mouse spleen lymphocytes (Subgroup B) were resuspended with PBS and anhydrous ethanol (1:3) and stored at −20 °C for 12 h. Then, the cells were washed twice with PBS and incubated with PI (#C0080; Solarbio, Beijing, China) for 30 min at 4 °C in the dark. The lymphocyte subset numbers, BrdU intensity, Ki67 expression, and cell cycling were then analyzed using a FACSAria TM III or FACS Calibur flow cytometer (BD Biosciences, San Jose, CA, USA). Data were analyzed using FlowJo software (V.10.8.1).

### 4.9. Western Blotting

Total protein from spleens was extracted using a RIPA lysis buffer (R0020; Solarbio, Beijing, China) with proteinase and phosphatase inhibitors (P1260; Solarbio, Beijing, China). The protein concentration was measured using a bicinchoninic acid (BCA) protein assay kit (BL521A; Biosharp, Beijing, China). An equal amount of protein (20–40 μg) was loaded into each well. After SDS-PAGE, proteins were transferred to a PVDF membrane (#10600023; Cytiva, Marlborough, MA, USA) and blocked with 5% non-fat milk for 1.5 h at room temperature. The membranes were incubated overnight at 4 °C with the following corresponding primary antibodies: anti-PCNA (1/1000; #13110; CST, Danvers, MA, USA), anti-P-JNK (1/1000; #4671; CST, Danvers, MA, USA), anti-JNK (1/1000; #9258; CST, Danvers, MA, USA), anti-P-p38 (1/1000; #9211; CST, Danvers, MA, USA), anti-p38 (1/1000; #9212; CST, Danvers, MA, USA), anti-P-ERK (1/1000; #4377; CST, Danvers, MA, USA), anti-ERK (1/1000; #4695; CST, Danvers, MA, USA), and anti-GAPDH (1/10000; #6004-1-Ig; Proteintech, Wuhan, China). After incubation, the membrane was washed 5 times and incubated with the secondary antibodies, including goat anti-rabbit IgG-HRP conjugate (1/10,000; ZB2301; ZSGB-BIO, Beijing, China) or goat anti-mouse IgG-HRP conjugate (1/10,000; ZB2205; ZSGB-BIO, Beijing, China) for 1 h at room temperature. Membranes were washed and detected using an enhanced chemiluminescent reagent kit (KF003; Affinity Biosciences, Cincinnati, OH, USA) and analyzed using Fiji software (version 2.16.0).

### 4.10. Statistical Analysis

The results were analyzed using GraphPad Prism version 8.04 (GraphPad Software, La Jolla, CA, USA) and expressed as the mean ± standard deviation (SD). Differences in mean values between groups were analyzed using a one-way ANOVA followed by Tukey’s multiple comparison test. For all statistical tests, a *p* value < 0.05 was considered to be statistically significant.

## 5. Conclusions

In conclusion, our study provides experimental evidence of PD on its immune regulatory function through activating the lymphocyte cycle and regulating thymic T cell development, which beneficially ameliorated CY-induced immunosuppression. These findings highlight PD’s potential as a novel therapeutic agent for immune-related disorders, particularly in contexts where immunosuppression is a significant concern, such as chemotherapy-induced immune suppression or aging-related immune decline.

However, several gaps remain to be addressed. For instance, the optimal dosing regimen and long-term safety profile of PD require further investigation. Additionally, future studies should explore PD’s effects on other models of immunosuppression, such as viral infections or autoimmune diseases, to fully understand its therapeutic potential. Comparative studies with other well-known immunomodulators, such as resveratrol and lentinan, could also provide valuable insights into PD’s unique advantages in terms of its efficacy, mechanism, and bioavailability.

In summary, this work not only advances our understanding of PD’s immunomodulatory mechanisms but also highlights its potential as a promising candidate for the treatment of immune-related disorders. By exploring PD’s effects on other immunosuppressive conditions, future research can further elucidate its therapeutic potential and pave the way for its clinical translation.

## Data Availability

The original contributions presented in this study are included in the article. Further inquiries can be directed to the corresponding author.

## Supplementary Materials

The following supporting information can be downloaded at: https://www.mdpi.com/article/10.3390/ijms26062800/s1.
