# Supplementary figures and images for: Polydatin Alleviates Cyclophosphamide-Induced Mouse Immunosuppression by Promoting Splenic Lymphocyte Proliferation and Thymic T Cell Development and Differentiation
Source: Int J Mol Sci. 2025 Mar 20;26(6):2800. doi: 10.3390/ijms26062800 (PMC11943104; doi:10.3390/ijms26062800)

Before selecting

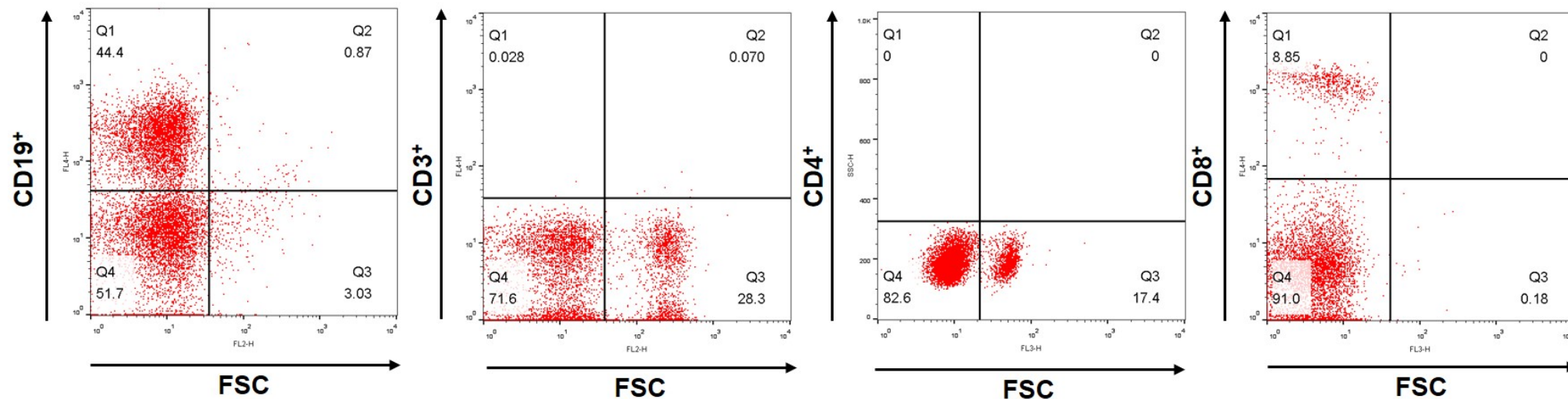

After selecting

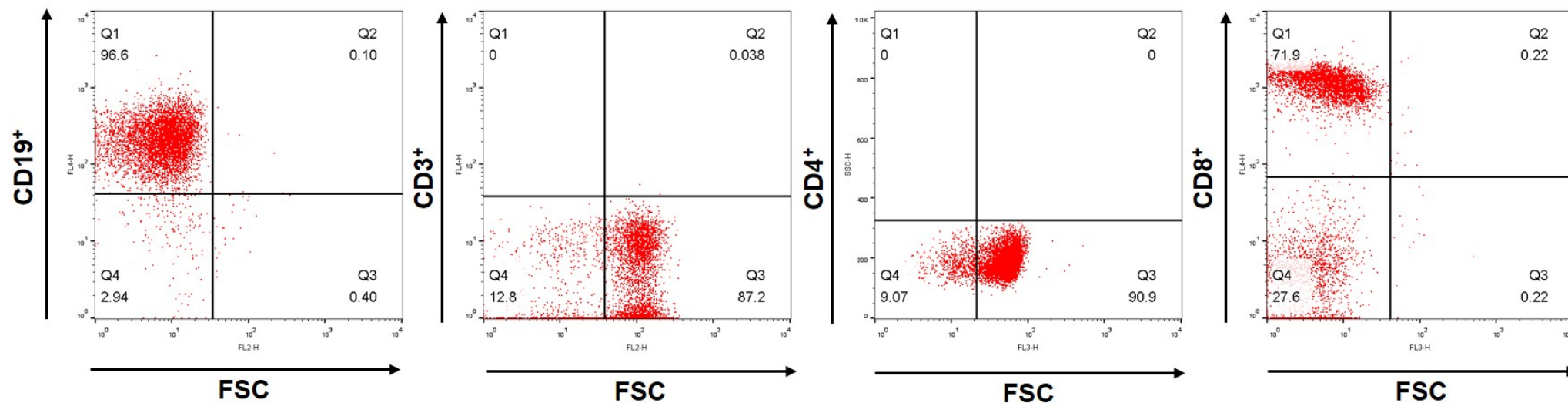

Original western blot (n=3)

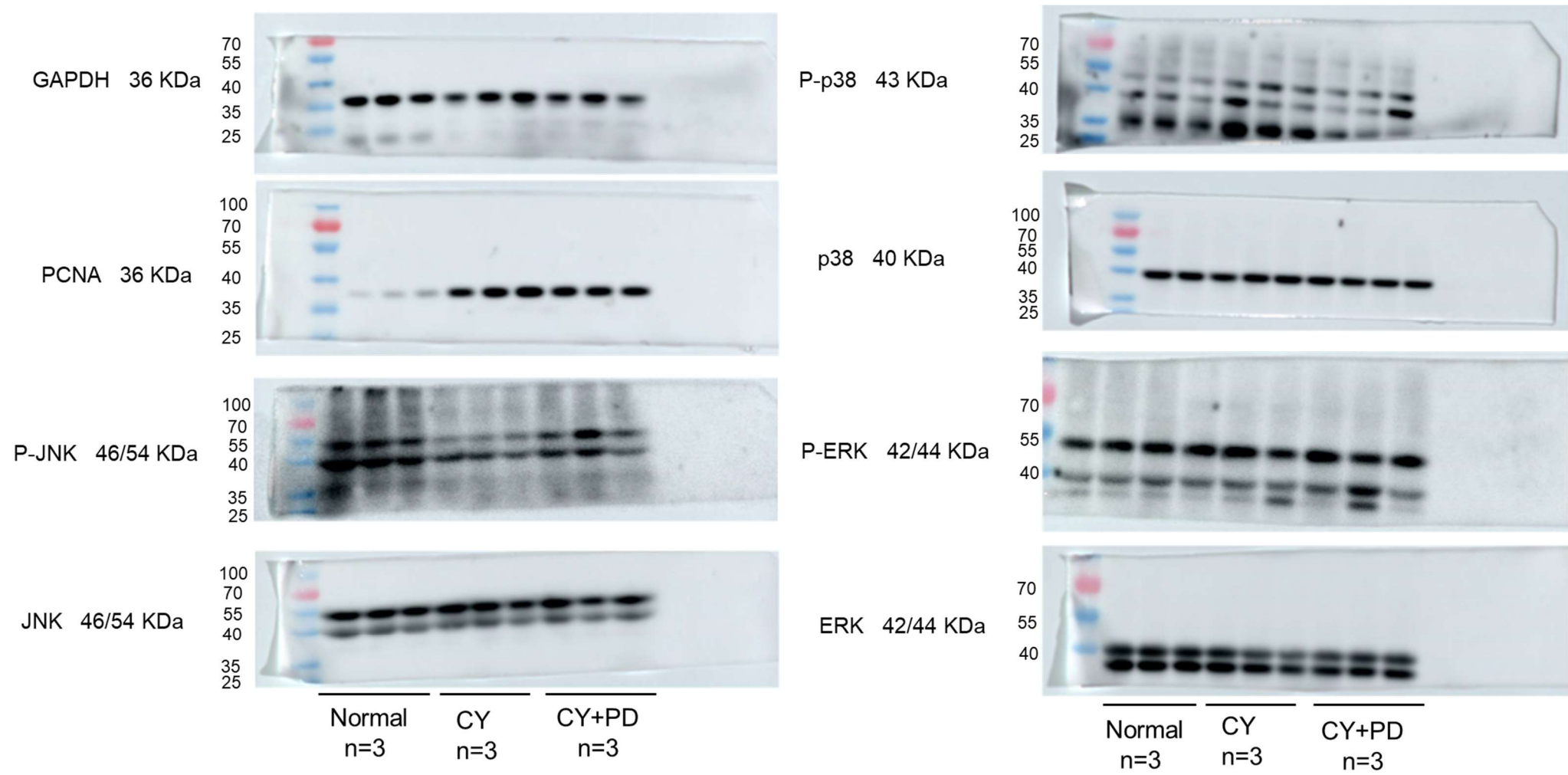

Supplement: Supplementary file 1 [file ijms-26-02800-s001.zip › ijms-3487296-supplementary.pdf]
